# Exploration of the Role of the Non-Coding RNA SbrE in *L. monocytogenes* Stress Response

**DOI:** 10.3390/ijms14010378

**Published:** 2012-12-24

**Authors:** Sana Mujahid, Teresa M. Bergholz, Haley F. Oliver, Kathryn J. Boor, Martin Wiedmann

**Affiliations:** 1 Department of Food Science, Cornell University, Ithaca, NY 14853, USA; E-Mails: sm832@cornell.edu (S.M.); tmb224@cornell.edu (T.M.B.); hfoliver@purdue.edu (H.F.O.); kjb4@cornell.edu (K.J.B.); 2 Department of Food Science, Purdue University, West Lafayette, IN 47907, USA

**Keywords:** non-coding RNA, *Listeria monocytogenes*, sigma B

## Abstract

SbrE is a ncRNA in *Listeria monocytogenes,* reported to be up-regulated by the alternative sigma factor σ^B^. Initial quantitative RT-PCR (qRT-PCR) experiments on parent strains and isogenic Δ*sigB* strains demonstrated σ^B^-dependent expression of SbrE across the four *L. monocytogenes* lineages and in *L. innocua*. Microarray and proteomics (MDLC/MS/MS with iTRAQ labeling) experiments with the *L. monocytogenes* parent strain and an isogenic Δ*sbrE* strain identified a single gene (*lmo0636*) and two proteins (Lmo0637 and Lmo2094) that showed lower expression levels in the Δ*sbrE* strain. qRT-PCR demonstrated an increase in SbrE transcript levels in stationary phase *L. monocytogenes* and in bacteria exposed to oxidative stress (mean log2 transcript levels 7.68 ± 0.57 and 1.70 ± 0.71 greater than in mid-log phase cells, respectively). However, no significant differences in growth or survival between the parent strain and Δ*sbrE* strain were confirmed under a variety of environmental stress conditions tested. Our data suggest that σ^B^-dependent transcription of SbrE represents a conserved mechanism that contributes, across *Listeria* species, to fine-tuning of gene expression under specific environmental conditions that remain to be defined.

## 1. Introduction

*Listeria monocytogenes* is a Gram-positive foodborne pathogen that causes listeriosis, a life threatening invasive illness in humans and animals [1]. *L. monocytogenes* is ubiquitous in the environment and has the ability to adapt to harsh and stressful conditions. For instance, the bacterium is able to grow at refrigeration temperatures and can survive high salt concentrations as well as acidic conditions [2–5]. This ability to adapt to harsh and stressful conditions facilitates *L. monocytogenes* survival under environmental, food, and host associated stress conditions. A complex transcriptional response network consisting of various signaling pathways and transcriptional regulators, including alternative sigma factors and non-coding RNAs (ncRNAs), supports the ability of *L. monocytogenes* to respond to and survive under a wide range of stress conditions [6–14].

ncRNAs have been shown to be involved in a variety of regulatory functions in bacteria, including regulation of bacterial response to stress and virulence related functions, through transcriptional, translational, and post-transcriptional regulation of gene expression [15–27]. In *L. monocytogenes*, more than 100 ncRNAs have been identified to date, including ncRNAs involved in virulence and stress response [7,11,14,28–34]. The alternative sigma factor Sigma B (σ^B^) is estimated to regulate transcription of 100 to 200 *L. monocytogenes* genes and contributes critically to the ability of this pathogen to survive stressful conditions encountered inside and outside the host [7,8,11–13]. σ^B^ is involved in the transcriptional response of *L. monocytogenes* to a variety of stresses, including osmotic and acid stress, as well as the regulation of metabolism and virulence [6,8,13]. *In vitro* and/or *in vivo* studies indicate that σ^B^ also directly regulates at least four ncRNAs in *L. monocytogenes* [7,11,30,31], in addition to possibly regulating ncRNAs indirectly by affecting transcription of *hfq*, which encodes a protein (Hfq) that binds to and regulates ncRNAs [7,11,27,35]. One σ^B^-dependent *L. monocytogenes* ncRNA is SbrE (also referred to as *rli47*), which was found to be highly transcribed in stationary phase cells using RNA-sequencing (RNA-Seq) [7]. A study using tiling arrays also found SbrE to be expressed at higher levels in stationary phase cells and in the intestinal lumen compared to exponential phase cells [11]. In addition, SbrE appears to be transcribed at higher levels in macrophages compared to exponential phase cells [36]. The 514 nucleotide sequence for SbrE is 96.6% conserved among 18 *L. monocytogenes* genomes, including EGD-e and F2365, and was found to be present in one *L. innocua* and one *L. welshimeri* genome [7]. In addition to identification of a putative σ^B^-dependent promoter upstream of SbrE, SbrE has been reported to show σ^B^-dependent transcript levels in *L. monocytogenes* strain 10403S [7] and EGD-e [11]. SbrE was also found to show σ^B^-dependent transcript levels in exponential phase cells and in *L. monocytogenes* present in the intestinal lumen, but not in *L. monocytogenes* inoculated into human blood [11]. As the role of SbrE has not yet been defined, we employed transcriptomic, proteomic, and phenotypic approaches to characterize the role of SbrE in σ^B^-dependent stress responses.

## 2. Results and Discussion

In this study, we demonstrate that (i) SbrE is σ^B^-dependent across *L. monocytogenes* lineages and in the non-pathogenic species *L. innocua*, and SbrE transcript levels are induced in stationary phase and under oxidative stress; (ii) SbrE contributes to the expression of an operon composed of *lmo0636* and *lmo0637*; (iii) contributions of SbrE to *L. monocytogenes* survival and growth under different stress conditions could not be identified, suggesting that SbrE may play a role in “fine-tuning of gene expression” in *L. monocytogenes*, which may only have phenotypic consequences under very specific growth conditions, as previously suggested for SbrA, another σ^B^-dependent ncRNA in *L. monocytogenes* [30].

### 2.1. SbrE Is σ^B^-Dependent Across *L. monocytogenes* Lineages and Induced in Stationary Phase and under Oxidative Stress

**Figure 1:**
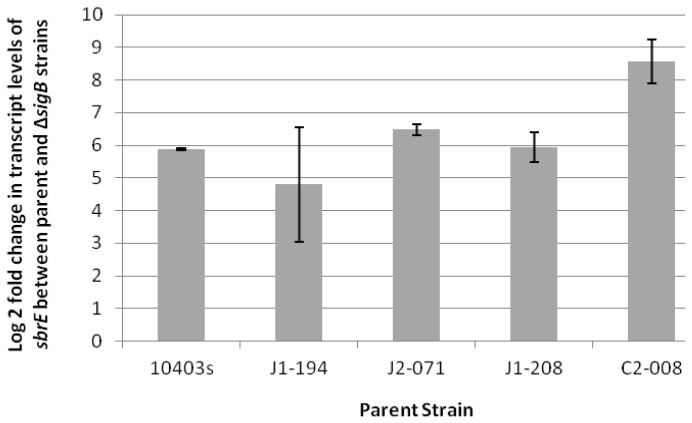
*sbrE* transcript levels detected using TaqMan qRT-PCR in parent strains relative to their Δ*sigB* null mutants. The y-axis shows the log2 fold change in *sbrE* transcript levels between parent strains and Δ*sigB* null mutant strains, calculated using the Pfaffl method. Data shown are mean values obtained from three independent experiments; error bars indicate standard deviation. The average log2 fold changes ranged from 4.8 to 8.6, which equals absolute (non-log transformed) fold changes of approximately 30 to 400.

qRT-PCR showed that, in stationary phase bacteria, SbrE transcript levels were significantly higher in parent strains relative to their Δ*sigB* mutants in (i) four strains representing all four *L. monocytogenes* lineages and (ii) an *L. innocua* strain (4.8 ± 1.76 to 8.6 ± 0.67 higher log2 SbrE transcript in the parent strain) (Figure 1), supporting σ^B^-dependent transcription of SbrE across *L. monocytogenes* lineages and in *L. innocua*. While these findings were not necessarily unexpected, they are still valuable as other studies have shown some diversification of the σ^B^ regulon and variation in σ^B^-dependent regulation of conserved genes, among *L. monocytogenes* lineages and *Listeria* species [10,37]. qRT-PCR of SbrE transcripts in the *L. monocytogenes* strain 10403S showed higher transcript levels in early stationary phase cells (OD 1.0 + 3h) as compared to mid-log phase (OD 0.4) or late log phase (OD 1.0) cells, consistent with σ^B^-dependent transcription of SbrE (as σ^B^ is induced in stationary phase cells) [7,11]. While SbrE transcript levels were not induced after exposure of mid-log phase cells to salt stress (Figure S1), they were induced after exposure to oxidative stress (13 mM cumene hydroperoxide [CHP]). SbrE transcript levels were 1.70 ± 0.71 log2 (absolute, non log-transformed fold change of approximately 3) higher in CHP treated cells, relative to mid-log phase cells.

### 2.2. SbrE Contributes to the Expression of an Operon Composed of *lmo0636* and *lmo0637*

Microarray experiments comparing transcript levels in *L. monocytogenes* 10403S parent and Δ*sbrE* strains identified a single gene that showed differential transcript levels (FC ≥ 1.5 and *p* ≤ 0.05). Specifically, *lmo0636* showed 2 fold lower transcript levels in Δ*sbrE* as compared to the parent strain. *lmo0636* transcript levels were also found to be downregulated in Δ*sigB*, as compared to the parent strain (FC = −2.17; *p* < 0.05). *lmo0636* transcript levels were not found to be significantly different in the microarray comparison between Δ*sigB* and Δ*sbrE.* qRT-PCR confirmed lower *lmo0636* transcript levels in Δ*sbrE* as compared to the parent strain (1.38 ± 0.16 log2 lower in Δ*sbrE; p* < 0.05 one sample *t*-test). These data indicate that *lmo0636* is positively regulated by SbrE.

Proteomics experiments identified two proteins that were differentially expressed (FC ≥ 1.2 and *p* < 0.05) between the *L. monocytogenes* parent strain and Δ*sbrE*. Lmo0637 and Lmo2094 both showed lower protein levels in the Δ*sbrE* strain (1.45 and 1.2 fold, respectively). Lmo2094 has been annotated as a metal ion binding, class II aldolase/adducin domain protein (Uniprot, www.uniprot.org). Lmo0637, annotated as an UbiE/COQ5 family methyltransferase, is encoded by a gene that forms a 2 gene operon with *lmo0636* [11]. Hence, the combination of transcriptional and proteomics results indicates that SbrE regulates the expression of the *lmo0636*-*lmo0637* operon. *lmo0636* encodes a protein that was annotated as a hypothetical 2Fe-2S cluster/DNA binding protein of the Rrf2 family of regulators, which belongs to the winged helix-turn-helix superfamily of transcriptional regulators [38]. The *N*-terminal and *C*-terminal regions of Rrf2 family proteins are generally involved in DNA binding and signaling, respectively, and may function as redox sensors [39]. Interestingly, previous studies were not able to identify *lmo0636*/*lmo0637* transcription patterns that would point towards a specific mechanism for regulation of this operon. While Raengpradub *et al*. [10] did not find *lmo0636* and *lmo0637* to be significantly differentially expressed in comparisons of *L. monocytogenes* 10403S and Δ*sigB*, in an L. monocytogenes prfA* genetic background (which expressed a constitutively active PrfA), both genes were found to have significantly higher transcript levels in Δ*sigB* strains, suggesting that they are negatively regulated by σ^B^ in the presence of an active PrfA [9]. On the other hand, *lmo0637* was found to be up-regulated in the host during mouse infection with *L. monocytogenes* EGD-e, as compared to stationary phase and exponential phase cells grown in BHI, while *lmo0636* was reported to be downregulated in the host as compared to stationary phase cells grown in BHI [40]. These data suggest that transcriptional regulation of *lmo0636*/*lmo0637* is highly dependent on environmental conditions and may be fine-tuned by SbrE and σ^B^-dependent transcription of *sbrE*.

**Figure 2:**
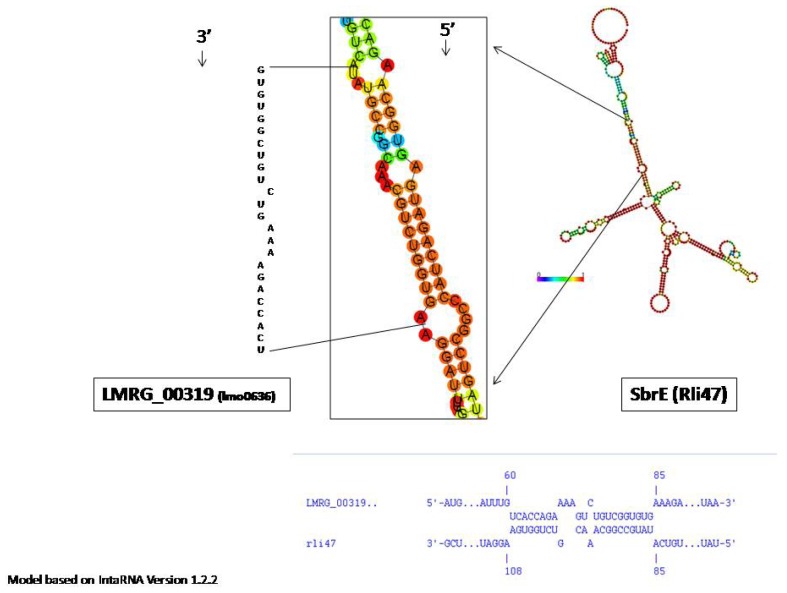
SbrE interaction with *LMRG_00319* (*lmo0636*) predicted using IntaRNA software. The ΔEnergy (kcal/mol) of the interaction is −11.75.

As *trans*-encoded ncRNAs largely act through base pairing with target RNAs, typically the 5′UTR, consequently affecting their translation and/or stability [27], we modeled the putative interaction between SbrE and lmo0636 *in silico*, using IntaRNA version 1.2.2 [41] (Figure 2). We only found an interaction with a ΔEnergy of −11.75 kcal/mol, indicating limited complementarily between SbrE and *lmo0636* (including its 5′UTR). A preliminary target capture experiment that used biotin-labeled SbrE bound to BioMag Streptavidin beads (Qiagen, Valencia, CA, USA) to capture *lmo0636* RNA (with subsequent detection by qRT-PCR) also found no evidence for a specific interaction between SbrE and lmo0636. Specifically, levels of *lmo0636* RNA recovered were not different from levels of RNA recovered for another *L. monocytogenes* gene with no evidence for SbrE dependent expression (*i.e.*, *lmo0514*). Future experiments are thus needed to identify the direct or indirect mechanism by which SbrE may influence the expression of the *lmo0636*-*lmo0637* operon or to identify other SbrE targets.

### 2.3. Contributions of SbrE to *L. monocytogenes* Survival and Growth under Different Stress Conditions could not Be Identified

As Lmo0636 is annotated as a protein that may play a role in oxidative stress response (see Section 2.2), we initially focused on characterizing the oxidative stress survival phenotype of the Δ*sbrE* mutant constructed here. Initial assays showed relative killing of 1.95 log CFU/mL for the Δ*sbrE* mutant as compared to 1.11 log CFU/mL for the parent strain after oxidative stress (13 mM CHP) exposure for 15 min, indicating a potentially small but significantly (*p* = 0.0084) reduced ability to survive oxidative stress for the Δ*sbrE* mutant (Figure S2).

As the difference in survival between the parent and the Δ*sbrE* strain was <1 log (*i.e.*, 0.84 ± 0.29 log CFU/mL), follow up experiments were conducted to monitor oxidative stress (13 mM CHP exposure) survival over 60 min. In these experiments, we found no significant differences in log reduction for the parent and the Δ*sbrE* strain after CHP exposure for 15, 30, and 60 min (*p* > 0.05), even though the Δ*sbrE* strain showed numerically higher log CFU reductions, as compared to the parent strain at each time point, with the difference being <1 log at each time point (Table 1 and Figure S3). We thus used a competitive index experiment, which provides a more sensitive approach to identify phenotypic differences between two strains, to compare the oxidative stress resistance between the parent and the Δ*sbrE* strain. After 13 mM CHP exposure for 15 min the competitive index comparing the Erm^r^ parent strain and the Erm^s^ Δ*sbrE* mutant was 1.63, virtually the same as for the control comparing the Erm^r^ parent strain to an Erm^s^ parent (1.50), suggesting no difference in oxidative stress survival between the parent and Δ*sbrE* strains in this experiment. In all three experiments detailed above we did find evidence for significantly reduced oxidative stress resistance of the Δ*sigB* strain, including a competitive index of 13.29 for the comparison between the parent strain and the Δ*sigB* strain. These findings are consistent with previous reports, which showed that σ^B^ contributes to oxidative stress resistance in *L. monocytogenes* [8].

**Figure 3:**
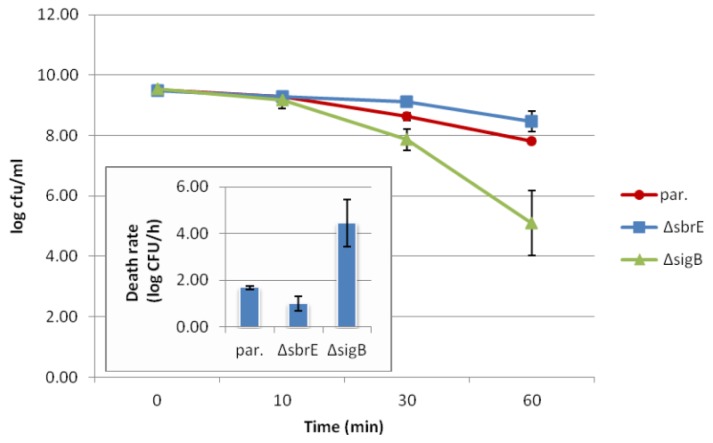
Acid stress survival of parent strain (par., circle), Δ*sbrE* (square), and Δ*sigB* (triangle) strains. Bacterial numbers in log Colony Forming Units per milliliter after exposure to pH 2.5 for 1 h are plotted. The inset shows the average death rate of each strain in log Colony Forming Units per hour. Values are means from three independent experiments; error bars indicate standard deviation.

Further phenotypic evaluation of the Δ*sbrE* strain showed no significant effect of the *sbrE* deletion on (i) ability to survive acid stress (pH 2.5, 1 h; see Table 1 and Figure 3), (ii) ability to survive under salt stress (1.75 M NaCl, 12 h; see Table 1 and Figure S4); (iii) growth under glucose-limiting conditions (0.04% wt/vol glucose, 30 h; see Table 1 and Figure S5), and (iii) growth at 7 °C for 12 days (Table 1 and Figure S6). On the other hand, the Δ*sigB* strain, which was included as a control, showed (i) significantly higher death rate under acid stress as compared to the parent strain (*p* = 0.0054) and Δ*sbrE* (*p* = 0.0022) (Table 1 and Figure 3); (ii) significantly reduced ability to survive salt stress as compared to the parent strain (*p* = 0.0039) and Δ*sbrE* (*p* = 0.0039) (Table 1 and Figure S4); and (iii) significantly greater increase in cell density under glucose limiting conditions as compared to the parent strain (*p* = 0.0008) and Δ*sbrE* (*p* = 0.0014) (Table 1 and Figure S5). The Δ*sigB* strain showed a small but significant (*p* = 0.0371) reduction in growth rate under cold stress compared to wildtype, with a difference of 0.06 ± 0.03 log CFU/mL/day (Table 1 and Figure S6). Susceptibility to infection from the 22 Listeriaphages tested did not differ between wildtype, Δ*sbrE*, and Δ*sigB* strains (Table S1).

Our data suggest that SbrE does not contribute to *L. monocytogenes* survival and growth under a number of stress conditions that are well established to require σ^B^ for optimal growth and survival. Overall, we found that a SbrE deletion does not affect *L. monocytogenes* growth under cold stress or energy stress or *L. monocytogenes* acid stress survival or phage resistance. SbrE did however show a small but significant contribution to the survival of *L. monocytogenes* 10403S under oxidative stress in our initial experiments; however, this phenotype was not confirmed by subsequent experiments. These data indicate potential contributions of *L. monocytogenes* SbrE to growth and survival under very specific and defined environmental stress conditions. Interestingly, the characterization of the σ^B^-dependent ncRNA SbrA also found no phenotypes for a Δ*sbrA* mutant strain under the conditions tested [30].

## 3. Experimental Section

### 3.1. Bacterial Strains and Growth Conditions

Strains used in this study are listed in Table 2. Stock cultures of all strains were stored at −80 °C in Brain Heart Infusion (BHI) medium containing 15% glycerol. Cultures were streaked onto BHI agar and incubated at 37 °C for 24 h to obtain isolated colonies for inoculation of overnight cultures. Specific growth conditions for each experiment are described below.

### 3.2. Construction of *L. monocytogenes* Mutants

A nonpolar internal deletion mutant allele of *sbrE* was created by splicing by overlap extension (SOE) PCR and allelic mutagenesis, using previously described procedures [44]. Allelic exchange mutagenesis of the wildtype *sbrE* allele with the mutant allele was confirmed by PCR amplification and direct sequencing of the PCR product (see Table S2 for primers).

### 3.3. TaqMan Quantitative RT-PCR (qRT-PCR) to Measure *sbrE* and *lmo0636* Transcript Levels

qRT-PCR was used to quantify (i) *sbrE* transcript levels in parent and Δ*sigB* mutant strains representing the different lineages of *L. monocytogenes* as well as one *L. innocua* strain, and (ii) *lmo0636* transcript levels in *L. monocytogenes* parent strain10403S and its isogenic Δ*sbrE* null mutant. Briefly, cells were grown to stationary phase at 37 °C as previously described [10], with shaking at 230 rpm. After cells reached stationary phase, RNAProtect bacterial reagent (Qiagen, Valencia, CA, USA) was used to stabilize the mRNA according to manufacturer’s instructions. Bacterial cells were collected by centrifugation and stored at −80 °C prior to RNA isolation. RNA extraction was performed using TRI reagent as described previously [45]. Total RNA was incubated with RNasin (Promega, Madison, WI, USA) and RQ1 DNase (Promega) to inhibit RNases and remove DNA contamination, respectively. Further RNA cleanup and concentration was performed using the RNeasy MinElute Cleanup Kit (Qiagen). A NanoDrop ND-1000 spectrophotometer (NanoDrop, Rockland, DE, USA) was used to quantify and assess purity of the RNA. RNA quality and integrity was assessed by the Agilent 2100 Bioanalyzer (Agilent, Santa Clara, CA, USA).

One microgram of RNA from each sample was reverse transcribed to cDNA using random hexamers and reverse transcriptase (TaqMan Reverse Transcription Reagents, Applied Biosystems, Carlsbad, CA, USA) prior to qRT-PCR. To evaluate residual genomic DNA contamination, control reactions without reverse transcriptase were included for each template. qRT-PCR was performed on an ABI Prism 7000 Sequence Detection System (Applied Biosystems), using the TaqMan Universal PCR Master Mix Reagent (Applied Biosystems). Duplicate qRT-PCR reactions were loaded into MicroAmp optical 96-well reaction plates and run using the following program: 1 cycle at 50 °C for 2 min, 1 cycle at 95 °C for 10 min, followed by 40 cycles at 95 °C for 15 s and 60 °C for 1 min. Standard curves for each target template were included to determine the amplification efficiency. All qRT-PCR analyses were performed in triplicate using RNA isolated from three independent biological replicates of cells (see Table S2 for primers and probes). Relative gene transcription levels, *i.e.*, fold changes, were calculated using the efficiency calibrated mathematical model described by Pfaffl [46]. Target transcript levels were normalized to transcript levels of the housekeeping gene *rpoB*, which displays relatively stable transcript levels under varying experimental conditions [8].

### 3.4. qRT-PCR to Determine Growth Phase and Environmental Stress Dependent sbrE Transcript Levels

qRT-PCR was used to measure *sbrE* transcript levels in mid-log phase (OD_600_ 0.4), late log phase (OD_600_ 1.0), early stationary phase (OD_600_ 1.0 + 3 h), and after exposure of mid-log phase cells to either (i) 13 mM cumene hydroperoxide (CHP) (Sigma-Aldrich, St. Louis, MO, USA), 15 min (as described by Oliver *et al*. [8]), or (ii) 10% NaCl, 15 min. *L. monocytogenes* 10403S cells were grown as described above. To apply salt stress, an equal volume inoculum of mid-log phase cells was transferred to 5 mL 20% NaCl, and cultures were then incubated at 37 °C with shaking for 15 min. RNA extraction, cDNA synthesis, and qRT-PCR were performed as described above, with the exception that RNA was purified using two phenol-chloroform extractions and one chloroform extraction, followed by RNA precipitation and resuspension in RNase-free water, instead of the RNeasy MinElute Cleanup Kit (Qiagen) procedure described above. Relative gene transcription levels were calculated using the Pfaffl model as described above [46] with target transcript levels normalized to transcript levels of *rpoB* within samples. Results from all samples were normalized to SbrE transcript levels from a single replicate at mid-log phase (OD 0.4) [47].

### 3.5. Microarray

*L. monocytogenes* 10403S parent strain, Δ*sbrE*, and Δ*sigB* cells were grown to stationary phase and total RNA was extracted as described above for qRT-PCR analysis. DNA Microarray design and construction were described in a previous study [10]. cDNA synthesis, labeling with dyes, and hybridization were performed as described by Ollinger *et al*. [9], with the exception that samples were labeled with Cy3 and Cy5 dyes (Amersham Biosciences, Piscataway, NJ, USA). Three replicates using three independent RNA isolations were performed for each microarray comparison (Δ*sbrE versus* parent strain; Δ*sbrE versus* Δ*sigB*). Microarray statistical analysis was performed as described previously [10]. A *n*-fold change of ≥1.5 was used as the cutoff for the identification of differentially expressed genes.

### 3.6. Protein Isolation, Digestion, and iTRAQ Labeling

*L. monocytogenes* 10403S and Δ*sbrE* were grown to stationary phase as described above. After growth to stationary phase, bacterial cells from 25 mL of culture were collected by centrifugation. Cell pellets were quick-frozen with liquid nitrogen and stored at −80 °C prior to protein isolation. Proteins were isolated as previously described [48] with slight modifications. Briefly, cell pellets were washed in 1 M triethyl ammonium bicarbonate buffer (pH 8.5) (Sigma-Aldrich, St. Louis, MO, USA) with 0.1% (wt/vol) SDS and 10 μg/mL chloramphenicol (extraction buffer (EB)). Cells were then lysed using a bead beater (BioSpec Mini-Beadbeater-1) in a mixture of 0.5 mm zirconia/silica beads and 1 mL EB. The protein sample was separated from beads by centrifugation and protein concentrations were determined using a noninterfering protein assay kit with bovine serum albumin as the standard (Calbiochem, San Diego, CA, USA) according to the manufacturer’s instructions. 1D SDS-PAGE was used to verify sample protein concentration and quality.

Protein samples were analyzed at the Cornell University Proteomics and Mass Spectrometry Core Facility using shotgun-based quantitative proteomics. A total of 100 μg protein of each sample was denatured, reduced with 5 mM tris-(2-carboxylethyl) phosphine at 37 °C for 1 h and the cysteine residues were blocked with 8 mM methyl methanethiosulfonate for 10 min at room temperature. Protein samples were digested with 10 μg of sequence-grade-modified trypsin at 37 °C for 16 h. Efficiency of protein digestion was assessed by SDS-PAGE. Tryptic peptides from *L. monocytogenes* parent strain 10403S and Δ*sbrE* were each labeled with iTRAQ reagents, according to the manufacturer’s protocols (document #4351918A and 4350831C downloaded from  docs.appliedbiosystems.com/search.taf; Applied Biosystems). The labeled samples were then combined and fractionated via Isoelectric focusing (IEF) OffGel electrophoresis (OGE) as described below.

### 3.7. OGE Fractionation and Nano-Scale Reverse Phase Chromatography and Tandem Mass Spectrometry (NanoLC-MS/MS)

The pooled iTRAQ labeled peptides were separated using an Agilent 3100 OFFGEL Fractionator (Agilent, G3100AA, Santa Clara, CA, USA) as described by Yang *et al*. [49]. Subsequent nanoLC-MS/MS was carried out using a LTQ-Orbitrap Velos (Thermo-Fisher Scientific, San Jose, CA, USA) mass spectrometer equipped with a nano-ion source as reported previously [49], with the Orbitrap Velos operated in positive ion mode with nano spray voltage set at 1.5 kV and source temperature at 175 °C.

### 3.8. Data Processing, Protein Identification, and Data Analysis

All MS and MS/MS raw spectra from iTRAQ experiments were processed using Proteome Discoverer 1.1 (PD1.1, Thermo) for subsequent database search using in-housed license Mascot Daemon (version 2.2.04, Matrix Science: Boston, MA, USA); quantitative processing, protein identification, and data analysis were conducted as described by Yang *et al*. [49], with some modifications. Briefly, the *L. monocytogenes* protein sequence database containing 4177 sequence entries downloaded from the Broad Institute (www.broadinstitute.org/annotation/genome/listeria_group/GenomesIndex.html) on 22 May, 2009 was used for database search. The default Mascot search settings included (i) one missed cleavage for full trypsin with fixed MMTS modification of cysteine, (ii) fixed 4-plex iTRAQ modifications on lysine and *N*-terminal amines, and (iii) variable modifications of methionine oxidation and 4-plex iTRAQ on tyrosine. The peptide mass tolerance and fragment mass tolerance values were 10 ppm and 30 mDa, respectively. To estimate the false discovery rate (FDR), an automatic decoy database search was performed in Mascot. The relative quantitation ratios were normalized (bias-corrected) using the “median ratio” procedure for the iTRAQ 4-plex in each set of experiments. Two biological replicates were analyzed independently.

The Wilcoxon signed rank test was applied to peptide ratios for each identified protein to determine significant changes between strains. The Fisher’s Combined Probability Test was used to combine FDR adjusted Wilcoxon *p*-values from each replicate into one test statistic for every protein to obtain a combined *p*-value. Proteins with peptide ratios exhibiting a Fisher’s Combined Probability Test *p*-value <0.05 and an iTRAQ protein ratio ≥1.2 in both replicates were considered significantly differentially expressed. Statistical analyses were conducted using R Statistical Software (version 2.14.0; The R Foundation for Statistical Computing: Vienna, Austria, 2011).

### 3.9. Determination of Acid and Oxidative Stress Resistance as well as Phage Resistance

Acid and oxidative stress survival of Δ*sbrE* was compared to Δ*sigB* and the 10403S parent strain. Cells were grown to stationary phase (OD_600_ of 1.0 + 3 h) as described above. For acid stress experiments, 12 N HCl was added to 5 ml aliquot of stationary phase cells to reduce the culture pH to 2.5 as described previously [8]. Bacterial cells were quantified at 10, 30 and 60 min after addition of HCl by plating on BHI agar using a spiral plater (Autoplate 4000; Spiral Biotech, Inc., Norwood, MA, USA). Three independent replicates were performed.

For oxidative stress experiments, 900 μL of stationary phase cells were exposed to 13 mM CHP for 15 min at 37 °C as described previously [8]. Bacterial numbers were quantified by plating as described above. Three independent replicates were performed. In separate experiments, stationary phase cells were also exposed to CHP as described above over a 60 min period, and bacterial numbers were quantified by plating as described above at 15 min, 30 min, and 60 min. At least three independent replicates were performed.

Survival of Δ*sbrE* and Δ*sigB* mutants after CHP stress was also examined using competitive-index experiments. Strains used for these experiments included the erythromycin sensitive parent strain 10403S (Erm^S^ 10403S) and an erythromycin-resistant 10403S derivative (DP-L3903; Erm^R^ 10403S). Oxidative stress exposure (13 mM CHP) was applied as described above to strains mixed in a 1:1 ratio including (i) Erm^S^ Δ*sbrE* and Erm^R^ 10403S and (ii) Erm^S^ Δ*sigB* and the Erm^R^ 1043S (to evaluate the relative survival of Δ*sbrE* and Δ*sigB* compared to 10403S). A control competition experiment was conducted with a 1:1 mixture of Erm^S^ 10403S and Erm^R^ 10403S. Competition experiments conducted with and without 13 mM CHP were plated on BHI and incubated at 37 °C for 24 h. A hundred colonies from BHI plates were patched onto BHI agar containing 1 μg erythromycin/ml (BHI-erm). For differential enumeration, total colonies on BHI-erm were subtracted from total colonies on BHI (100). The competitive index was then calculated as the ratio of Erm^R^ to Erm^S^ colonies [43,50]. *L. monocytogenes* 10403S as well as Δ*sbrE* and Δ*sigB* strains were also tested for resistance against 22 diverse listeriaphages, using the procedures described by Vongkamjan *et al*. [51].

### 3.10. Salt, Cold, and Energy Stress Growth Experiments

Growth of 10403S parent strain, Δ*sbrE*, and Δ*sigB* strains under salt, cold, and energy stress conditions was compared. For salt growth experiments, cells were grown to mid-log phase (OD_600_ of 0.4) as described above. A 0.01% inoculum (vol/vol) was transferred to 50 mL pre-warmed BHI broth supplemented with 1.75 M NaCl in a 300 mL nephelo flask (5 μL into 50 mL). Cells were then incubated for 48 h at 37 °C with shaking (230 rpm). Cell numbers were determined, by plating on BHI agar, at specific time points over 48 h.

For cold growth experiments cells were grown to mid-log phase (OD_600_ of 0.4) as described above. A 0.01% inoculum (vol/vol) was transferred to pre-chilled 50 mL BHI broth in a 300 mL nephelo flask. Cells were then incubated at 7 °C for 12 days without shaking, and cell numbers were determined by plating on BHI agar using a spiral plater. For energy stress experiments, carbon starvation was induced by growing cells in defined medium (DM) containing a growth-limiting concentration of glucose (0.04%, wt/vol) [52]. Cells were initially grown in 5 mL of BHI broth at 37 °C overnight with shaking (230 rpm). A 0.1 mL aliquot of the overnight culture was inoculated into 10 mL DM supplemented with 0.4% (wt/vol) glucose and incubated for 12 h with shaking (230 rpm), followed by inoculation of a 0.01 mL aliquot into 10 mL pre-warmed DM containing 0.04% glucose and subsequent incubation for 30 h at 37 °C with aeration. Cell numbers were determined by plating on BHI agar using a spiral plater at specific time points over 30 h. Three biological replicates were performed for each growth experiment.

### 3.11. Statistical Analyses of Stress Experiments

The Baranyi model [53] was used to estimate maximum growth rates (μ_max_) for cold stress experiments, using the NLStools package (version 0.0-5) in R v2.6.2 (The R Foundation for Statistical Computing: Vienna, Austria, 2008). All other statistical analyses were performed with the Statistical Analysis Software (SAS) (version 9.0; SAS Institute, Inc. Cary, NC, USA, 2002). Regression analysis was used to calculate the death rate of cells exposed to pH 2.5, which was expressed as average log CFU death per hour for each strain. Analysis of variance (ANOVA) was used to test if there was significant difference in the death rates between the parent strain (10403S), Δ*sbrE*, and Δ*sigB*. ANOVA was also used to test for (i) differences in cell death due to oxidative stress; (ii) differences in growth rate (μmax) of cells exposed to cold stress, which was expressed as increase in cell density in log10 CFU/mL per day; (iii) differences in bacterial numbers after exposure to salt stress and growth under energy stress. Significance was set at *p* < 0.05 for all statistical analyses.

## 4. Conclusions

Our work showed that SbrE is a conserved part of the σ^B^ regulon, being σ^B^-dependent across *L. monocytogenes* lineages and in *L. innocua*. A combination of proteomics and microarray approaches indicates that SbrE contributes to regulating the expression of an operon composed of *lmo0636* and *lmo0637*, which encodes two proteins annotated as a hypothetical 2Fe-2S cluster/DNA binding protein and methyltransferase, respectively. SbrE dependent regulation of this operon likely occurs directly or indirectly at the mRNA level through the regulation of transcription or mRNA stability, as both microarray and qRT-PCR showed lower transcript levels for *lmo0636* in the Δ*sbrE* strain. While transcription of *sbrE* was found to be induced under oxidative stress conditions, phenotypic data could not find consistent evidence for contributions of SbrE to oxidative stress resistance, even though a trend towards reduced oxidative stress resistance was found in some experiments. As no phenotypic consequences of a *sbrE* deletion were found for environmental stress conditions under which σ^B^ had previously been demonstrated to be important for survival or growth, we hypothesize that SbrE may play a role in a “fine-tuning of gene expression” in *L. monocytogenes* or that it may play a role for *L. monocytogenes* fitness under very specific growth conditions that were not tested here as previously proposed by Nielsen *et al*. [30] for SbrA, another σ^B^-dependent ncRNA. Our data thus support that in addition to playing a role as a major regulator of certain stress response pathways (e.g., acid stress), σ^B^ is also likely to contribute more subtly to *L. monocytogenes* adaptation to other environmental stress conditions, including through complex regulatory networks. Additional experiments that utilize overexpression of SbrE will be needed, however, to gain further insight into the role of SbrE in *L. monocytogenes*.

## Supplementary Information



## Figures and Tables

**Table 1:** Environmental stress survival and growth of *L. monocytogenes* 10403S parent strain, Δ*sbrE*, and Δ*sigB*.

Reduction in cell numbers (log CFU/mL) after oxidative stress (13 mM CHP)^a^				Death rate (log CFU/h) after acid stress (pH 2.5 for 1 h) a	Average μmax (log CFU/mL/day) at 7 °C a	Increase in cell density (log CFU/mL) over 27 h growth in DM/0.04% glucose (Energy Stress) a,b	Cell numbers (log CFU/mL) after 12 h of growth in BHI with 1.75 M NaCl (Salt Stress) a
Strain	15 min	30 min	60 min				
Parent strain	2.46 ± 0.36	2.74 ± 0.08	2.69 ± 0.24	1.70 ± 0.08	0.73 ± 0.02	0.53 ± 0.04	4.86 ± 0.07
Δ*sbrE*	2.75 ± 0.62	3.02 ± 0.05	3.39 ± 0.13	1.01 ± 0.32	0.68 ± 0.01	0.64 ± 0.17	4.61 ± 0.11
Δ*sigB*	3.99 ± 0.88	3.62 ± 0.15 #,^	4.76 ± 0.20 #,^	4.45 ± 1.02 #,^	0.67 ± 0.01 #	1.45 ± 0.06 #,^	3.82 ± 0.12 #,^

a Data shown are means of at least three biological replicates ± standard deviation;

# Indicates significant difference between the parent strain and ΔsigB;

^ Indicates significant difference between ΔsigB and ΔsbrE;

b Increase in cell density was calculated as Colony Forming Units per milliliter after 30 h in DM minus Colony Forming Units per milliliter after 3 h in DM.

**Table 2:** Strains used in this study.

Strain	Lineage	Serotype	Origin
10403S	II	1/2a	Laboratory type strain
FSL A1-254, *ΔsigB*	II	1/2a	10403S (Wiedmann *et al*. [42])
FSL B2-236, *ΔsbrE*	II	1/2a	10403S
FSL J1-194	I	1/2b	Human clinical case
FSL C6-001, *ΔsigB*	I	1/2b	FSL J1-194 (Oliver *et al*. [8])
FSL J2-071	IIIA	4c	Bovine clinical case
FSL O1-006, *ΔsigB*	IIIA	4c	FSL J2-071 (Oliver *et al*. [8])
FSL J1-208	IV	4a	Caprine clinical case
FSL O1-005, *ΔsigB*	IV	4a	FSL J1-208 (Oliver *et al.* [8])
FSL C2-008			*L. innocua* DD 680
FSL R4-009, *ΔsigB*			*L. innocua* DD 680 **(**Raengpradub *et al.* [10])
DP-L3903, Erm^r^			10403S (Auerbuch *et al.* [43])
