# Correction: Wiedmann, M., *et al*. Exploration of the Role of the Non-Coding RNA SbrE in *L. monocytogenes* Stress Response. *Int. J. Mol. Sci.* 2013, *14*, 378–393

**DOI:** 10.3390/ijms14059685

**Published:** 2013-05-03

**Authors:** Sana Mujahid, Teresa M. Bergholz, Haley F. Oliver, Kathryn J. Boor, Martin Wiedmann

**Affiliations:** 1 Department of Food Science, Cornell University, Ithaca, NY 14853, USA; E-Mails: sm832@cornell.edu (S.M.); tmb224@cornell.edu (T.M.B.); hfoliver@purdue.edu (H.F.O.); kjb4@cornell.edu (K.J.B.); 2 Department of Food Science, Purdue University, West Lafayette, IN 47907, USA

The original version of the paper in Section 3.8 reports that *“The peptide mass tolerance and fragment mass tolerance values were 10 ppm and 30 mDa, respectively”* [1] (p. 387). To help others who may want to use the same methods in the future, the authors would like to correct the wording to: “*The peptide mass tolerance and fragment mass tolerance values were 30 ppm and 0.15 Da, respectively. In order to decrease the probability of false peptide identification, only peptides with significance scores above the identity threshold (at the 95% confidence interval), defined by Mascot probability analysis (www.matrixscience.com/help/scoring_help.html#PBM), were considered to be confidently identified and used for protein identification. Furthermore, only proteins identified in all four iTRAQ samples through at least two peptides with a p-value of <0.05 (expectation value) were further analyzed*”. The authors would like to apologize for any inconvenience this may have caused to the readers of this journal.

## References

[1] Mujahid S., Bergholz T.M., Oliver H.F., Boor K.J., Wiedmann M. (2013). Exploration of the role of the non-coding RNA SbrE in *L. monocytogenes* stress response. Int. J. Mol. Sci.

