# Supplementary material for: Exploration of the Role of the Non-Coding RNA SbrE in L. monocytogenes Stress Response
Source: Int J Mol Sci. 2012 Dec 24;14(1):378–93. doi: 10.3390/ijms14010378 (PMC3565269; doi:10.3390/ijms14010378)
Supplement: Supplementary file 1 [file ijms-14-00378-s001.pdf]

# Supplementary Information

**Figure S1.** Growth phase and environmental stress dependent expression of SbrE. Mean log2 transcript levels of SbrE at late log phase (OD 1.0), early stationary phase (OD 1.0 + 3 h), as well as after exposure of mid-log phase cells to CHP (13 mM cumene hydroperoxide, 15 min) or salt (10% NaCl, 15 min) stress relative to mid-log phase (OD 0.4) are indicated. Values are means from three independent qRT-PCR experiments; error bars indicate standard deviation.

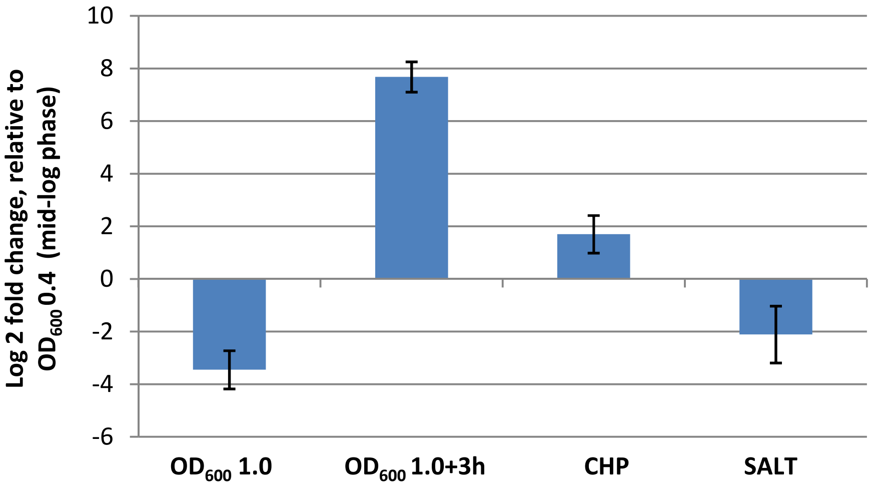


**Figure S2.** Oxidative stress survival of parent, Δ*sigB*, and Δ*sbrE* strains. Bacterial numbers in log Colony Forming Units per milliliter before and after exposure to 13 mM CHP stress for 15 min are shown. Survival was expressed as log reduction in viable cells, which was calculated by subtracting bacterial numbers of stressed cells from non-stressed controls. The difference in cell death (in log Colony Forming Units per milliliter) between strains is indicated. Values are means from three independent experiments; error bars indicate standard deviation.

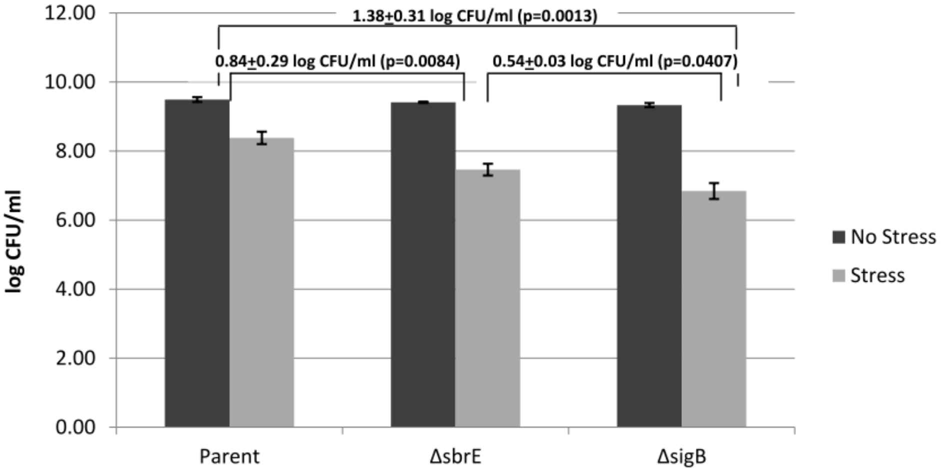

**Figure S3.** Oxidative stress survival of parent (par., circle), Δ*sbrE* (square), and Δ*sigB* (triangle) strains over 60 min. Reduction in cell numbers in log Colony Forming Units per milliliter after exposure to 13 mM CHP for 15 min, 30 min, and 60 min is shown. Values are means from at least three independent experiments; error bars indicate standard deviation.

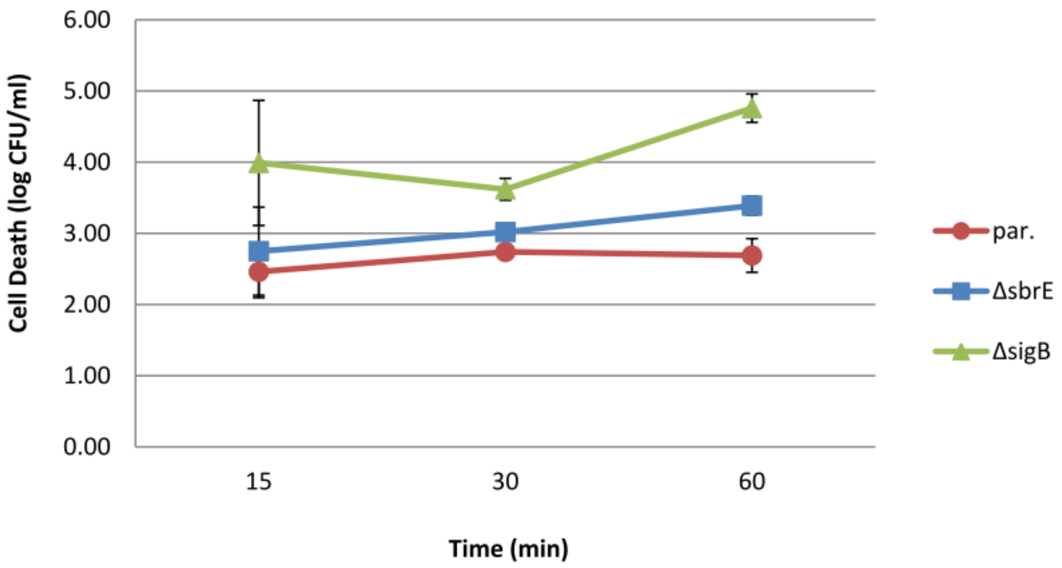


**Figure S4.** Salt stress survival of parent strain (par., circle), Δ*sbrE* (square), and Δ*sigB* (triangle) strains. Bacterial numbers in log Colony Forming Units per milliliter over 48 h of growth in BHI supplemented with 1.75 M NaCl are plotted. The inset shows the decrease in cell density of each strain from 0–12 h. Values are means from three independent experiments; error bars indicate standard deviation.

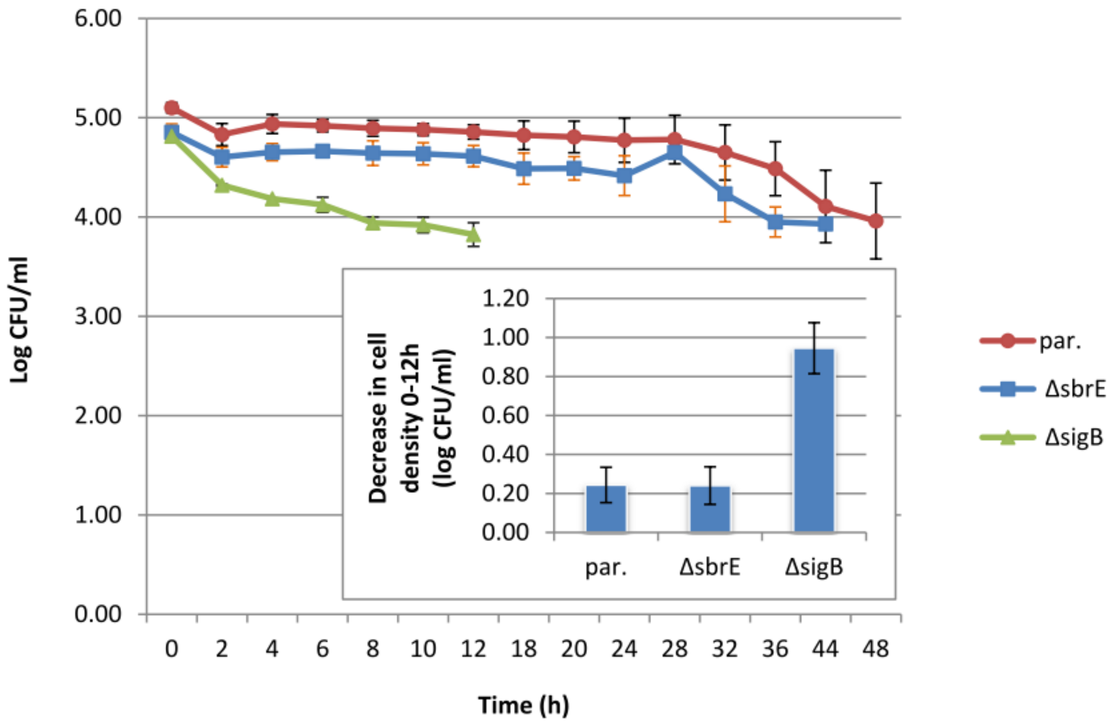

**Figure S5.** Growth of parent strain (par., circle), Δ*sbrE* (square), and Δ*sigB* (triangle) strains under energy stress. Bacterial numbers in log Colony Forming Units per milliliter over 30 h of growth in glucose-limiting DM are plotted. The inset shows the increase in cell density of each strain from 3–30 h. Values are means from three independent experiments; error bars indicate standard deviation.

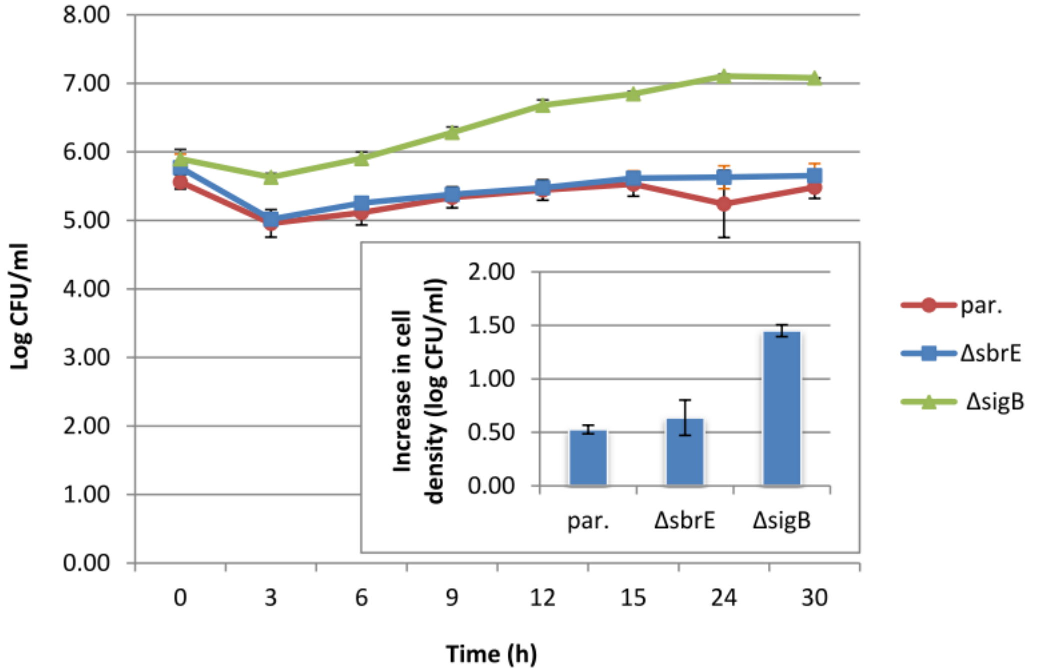


**Figure S6.** Growth of parent strain (par., circle), Δ*sbrE* (square), and Δ*sigB* (triangle) under cold stress (7 °C). Bacterial numbers in log Colony Forming Units per milliliter over 12 days of growth are plotted. The inset shows the average growth rate (μmax) of each strain in log Colony Forming Units per milliliter per day. Values are means from three independent experiments; error bars indicate standard deviation.

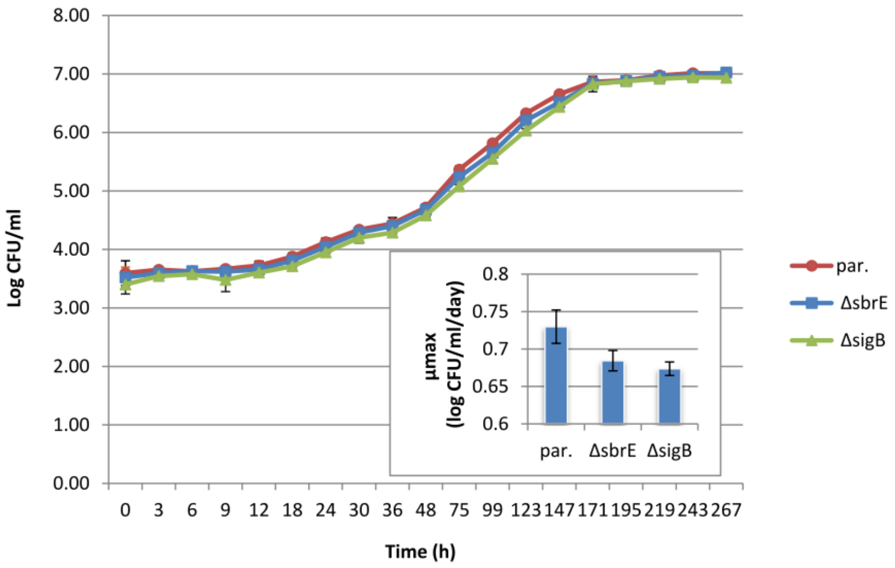

**Table S1.** Comparison of plaque formation in *L. monocytogenes* 10403S, Δ*sbrE*, Δ*sigB*, and MACK after infection with phages.

| Phage | 10403S [a] | Δ*sigB* [a] | Δ*sbrE* [a] | MACK [a] |
|---|---|---|---|---|
| LP-047 | - | - | - | - |
| LP-106 | +++ | +++ | +++ | +++ |
| LP-044 | +++ | +++ | +++ | +++ |
| LP-034 | +++ | +++ | +++ | ++ |
| LP-095 | +++ | +++ | +++ | +++ |
| LP-038 | ++ | ++ | +++ | +++ |
| LP-054 | - | - | - | - |
| LP-103 | +++ | +++ | +++ | +++ |
| LP-110 | +++ | +++ | +++ | +++ |
| LP-049 | ++ | ++ | ++ | +++ |
| LP-048 | +++ | +++ | +++ | +++ |
| LP-083 | NA | NA | NA | NA |
| LP-101 | - | - | - | +++ |
| LP-014 | - | - | - | - |
| LP-017 | + | + | + | + |
| LP-020 | - | - | - | - |
| LP-021 | - | - | - | - |
| LP-030 | - | - | - | - |
| LP-090 | +++ | +++ | +++ | +++ |
| LP-099 | +++ | +++ | +++ | +++ |
| LP-109 | +++ | +++ | +++ | +++ |
| LP-114 | +++ | +++ | +++ | +++ |

[a] Plaque formation: - indicates no plaque; + indicates turbid plaque; ++ indicates diffuse plaque (incomplete lysis); +++ indicates clear plaque (confluent lysis).

**Table S2.** Primers and probes used in this study.

| Primer | Sequence (5'→3') |
|---|---|
| SOE-A [a] | CGTCTAGAGCAGAAATTGATATCGCTGTGC |
| SOE-B | GCATCACCTTTTATTTGTTCGTTGTAC |
| SOE-C [b] | GTACAACGAACAAATAAAAGGTGATGCGGCAAGACAAGCTCATCCG |
| SOE-D [c] | CGAAGCTTACTAGCTGCTCGAGAGCATG |
| SbrE-XF | ACTTAAAAGTCCGCCCGG |
| SbrE-XR | ACAAAAACTACAAGAACAAGACGCAG |
| SbrE-Fwd | CAGGAGGAAGGCGAGGAGTATA |
| SbrE-Rev | CGATACTTTATTCGCTTATTTACCAATG |
| SbrE-probe | CGG AAT TTC GTT ACG TCG C |
| rpoB-Fwd | CCGGACGTCACGGTAACAA |
| rpoB-Rev | CAGGTGTTCCGTCTGGCATA |

**Table S2.** *Cont.*

| | |
|---|---|
| rpoB-probe [d] | TTATCTCCCGTATTTTACC |
| lmo0636-Fwd | ACCCTAAAAACCACAGCGAAAG |
| lmo0636-Rev | CCTTATTCATCACTTCGCCAATC |
| lmo0636-probe [d] | CAGCCGCACTGCT |

[a] The *Xba*I restriction site incorporated into this primer to facilitate cloning is underlined; [b] The overhang complementary to SOE-B is underlined; [c] The *Hind*III restriction site incorporated into this primer to facilitate cloning is underlined; [d] TaqMan probes were designed with FAM-5' and MGB-3' ends.

© 2013 by the authors; licensee MDPI, Basel, Switzerland. This article is an open access article distributed under the terms and conditions of the Creative Commons Attribution license (http://creativecommons.org/licenses/by/3.0/).
